# Clinical and Laboratory Predictors for the Development of Low Cardiac Output Syndrome in Infants Undergoing Cardiopulmonary Bypass: A Pilot Study

**DOI:** 10.3390/jcm10040712

**Published:** 2021-02-11

**Authors:** Sarah E. Drennan, Kathryn Y. Burge, Edgardo G. Szyld, Jeffrey V. Eckert, Arshid M. Mir, Andrew K. Gormley, Randall M. Schwartz, Suanne M. Daves, Jess L. Thompson, Harold M. Burkhart, Hala Chaaban

**Affiliations:** 1 Section of Neonatal-Perinatal Medicine, Department of Pediatrics, University of Oklahoma Health Sciences Center, Oklahoma City, OK 73104, USA; sarah.drennan@live.com (S.E.D.); kathryn-burge@ouhsc.edu (K.Y.B.); edgardo-szyld@ouhsc.edu (E.G.S.); jeffrey-eckert@ouhsc.edu (J.V.E.); 2 Section of Cardiology, Department of Pediatrics, University of Oklahoma Health Sciences Center, Oklahoma City, OK 73104, USA; arshid-mir@ouhsc.edu; 3 Section of Pediatric Critical Care, Department of Pediatrics, University of Oklahoma Health Sciences Center, Oklahoma City, OK 73104, USA; andrew-gormley@ouhsc.edu; 4 Department of Anesthesiology, University of Oklahoma Health Sciences Center, Oklahoma City, OK 73104, USA; randall-schwartz@ouhsc.edu (R.M.S.); suanne-daves@ouhsc.edu (S.M.D.); 5 Department of Cardiovascular Surgery, University of Oklahoma Health Sciences Center, Oklahoma City, OK 73104, USA; jess-thompson@ouhsc.edu (J.L.T.); harold-burkhart@ouhsc.edu (H.M.B.)

**Keywords:** low cardiac output syndrome, cardiopulmonary bypass, inflammation, congenital heart disease, pediatric cardiology

## Abstract

Cardiac surgery employing cardiopulmonary bypass exposes infants to a high risk of morbidity and mortality. The objective of this study was to assess the utility of clinical and laboratory variables to predict the development of low cardiac output syndrome, a frequent complication following cardiac surgery in infants. We performed a prospective observational study in the pediatric cardiovascular ICU in an academic children’s hospital. Thirty-one patients with congenital heart disease were included. Serum levels of nucleosomes and a panel of 20 cytokines were measured at six time points in the perioperative period. Cardiopulmonary bypass patients were characterized by increased levels of interleukin-10, -6, and -1α upon admission to the ICU compared to non-bypass cardiac patients. Patients developing low cardiac output syndrome endured longer aortic cross-clamp time and required greater inotropic support at 12 h postoperatively compared to bypass patients not developing the condition. Higher preoperative interleukin-10 levels and 24 h postoperative interleukin-8 levels were associated with low cardiac output syndrome. Receiver operating characteristic curve analysis demonstrated a moderate capability of aortic cross-clamp duration to predict low cardiac output syndrome but not IL-8. In conclusion, low cardiac output syndrome was best predicted in our patient population by the surgical metric of aortic cross-clamp duration.

## 1. Introduction

Low cardiac output syndrome (LCOS) is a serious and frequent complication of cardiopulmonary bypass (CPB) in infants following cardiac surgery [1]. LCOS refers to a postoperative state of reduced cardiac output, which may lead to multi-organ system failure and death [2]. Predicting which patients will develop LCOS remains difficult, and treatment at this time is limited to timely supportive care, often resulting in a greater duration of postoperative mechanical ventilation, prolonged cardiovascular intensive care unit (CVICU) length of stay (LOS), and more interventions for cardiac support [3,4].

The etiology of LCOS is generally considered to be a multifactorial aggregate of factors related to both anatomical and physiological characteristics of the heart defect under repair and additional physiological stress incurred during the surgery, including oxidative stress [5] through the use of CPB and ischemia/reperfusion injuries of the heart and lungs [1]. Most of these etiological factors induce systemic inflammation, thought to be a major driver of several postoperative morbidities, including LCOS [6,7]. Pediatric cardiac surgeons utilizing CPB routinely attempt to dampen associated postoperative systemic inflammation through a variety of mechanisms, including administration of perioperative steroids [8], ultrafiltration, and the use of heparin [9]. Postoperative systemic inflammation is of particular concern in neonates, an age group characterized by comparatively hyperinflammatory responses [10]. Previous studies have reported elevated cytokine levels, both inflammatory and anti-inflammatory [7,9,11,12,13], in infants following CPB.

Nucleosomes, complexes of DNA bound with histones, are either released during cell death or secreted following cell damage or inflammation [14]. These complexes are a marker of neutrophil extracellular traps (NETs), web-like structures released by neutrophils to ensnare potential pathogens. NETs are implicated in, or associated with, the pathophysiology of several critical conditions, including the use of CPB during adult cardiac surgery [15]. Elevated nucleosome levels have been noted following myocardial ischemia-reperfusion injury [16], and prolonged elevations in extracellular DNA [17] or rapidly peaking levels of circulating histones [18] have been correlated to poor postoperative outcomes following cardiac surgery in adults and young children, respectively. Whether nucleosome release during CPB is associated with the development of LCOS, however, is unknown. Few studies investigating clinical or laboratory correlates of poor outcomes following cardiac surgery with CPB have included the development of LCOS as an outcome of interest.

In this study, the hypothesis that histones and DNA (nucleosomes) are released in pediatric cardiac patients who undergo cardiopulmonary bypass, and are superior in the early detection of LCOS compared to cytokine/chemokine levels, was investigated. Thus, both surgical characteristics and laboratory levels of circulating nucleosomes and cytokines during the perioperative period in a control group of infants undergoing surgical repair of congenital heart disease (CHD) without CPB were compared to those of a similar group of surgical infants requiring cardiopulmonary bypass. Importantly, the potential correlations of these variables with the development of LCOS in bypass patients were investigated. Finally, the ability of these surgical or laboratory metrics to predict the development of LCOS was examined in an attempt to identify early biomarkers or therapeutic targets for the condition.

## 2. Materials and Methods

### 2.1. Study Design

This prospective observational study was approved by the Institutional Review Board at the University of Oklahoma Health Sciences Center (IRB #7676, approved 03/10/2017; ClinicalTrials.gov Identifier NCT03143348). Due to the lack of previous data on nucleosome levels as predictors of LCOS, the study was designed as a convenience sample pilot study, and sample size calculation was not performed. Written informed consent was obtained from one or both parents. Thirty-two infants with CHD confirmed postnatally, born at or above 36 weeks gestational age, with a birth weight greater than 2.5 kg, and less than 6 months old, were enrolled from June 2017 through November 2018 (Figure S1 in Supplementary Materials). Infants were excluded from the study due to the following: an expectation of increased inflammatory markers before the study (e.g., requiring two or more vasopressors); preoperative proven sepsis or cardiac catheterization within one week of surgical date; previous surgery except for pulmonary artery banding; or presence of significant extra-cardiac anomalies thought to impair organ function, including congenital renal anomalies, airway anomalies, and gastrointestinal anomalies such as imperforate anus and intestinal atresia.

Infants were enrolled into one of two groups based on the anatomy of cardiac lesions. Group 1 was composed of patients with CHD requiring cardiac surgery without CPB (e.g., coarctation of the aorta (COA) and pulmonary arterial bands for tricuspid atresia with normally related great arteries (TA/NRGA)). Group 2 constituted CHD patients requiring cardiac surgery with CPB (severe Group 1 anomalies, in addition to hypoplastic left heart syndrome (HLHS), interrupted aortic arch (IAA), atrial septal defect (ASD), double outlet right ventricle (DORV), pulmonary atresia (PA), dextro-transposition of the great arteries (D-TGA), aortic valve stenosis (AS), and tetralogy of Fallot (TOF)). Demographic and perioperative data were compared between groups, as well as stratified in Group 2 patients, by the development of LCOS.

The short-term risk of mortality was estimated via the six-category RACHS-1 (Risk Adjustment for Congenital Heart Surgery) model and the European Association for Cardiothoracic Surgery (EACTS) and the Society of Thoracic Surgeons (STS) mortality categories (EACTS-STS mortality Categories) [19]. Mortality risk assessments at CVICU admission were estimated by both PIM2 (Pediatric Index of Mortality 2) [20] and PRISM III (Pediatric Risk of Mortality III) [21] models, using information collected during the first hour (PIM2) or the first 12 h and 24 h (PRISM III) in the CVICU. Morbidity and mortality in the CVICU were also estimated serially with the Sequential Organ Failure Assessment (SOFA) score [22]. The vasoactive-inotropic score (VIS) was recorded at CVICU admission and 12 h and 24 h into CVICU stay. VIS, a surrogate measure for morbidity and mortality following cardiac surgery, was calculated as dopamine dose (µg/kg/min) + dobutamine dose (µg/kg/min) + (100 × epinephrine dose (µg/kg/min)) + (10 × milrinone dose (µg/kg/min)) + (10,000 × vasopressin dose (U/kg/min)) + (100 × norepinephrine dose (µg/kg/min)) [23].

Diagnosis of LCOS, the primary outcome, was based on guidelines modified from Carmona et al. [7], consisting of at least two of the following criteria at any postoperative timepoint: prolonged capillary refill >3 s, systolic blood pressure <5th percentile for age and gender, low urine output (<1 cc/kg/h) for at least 6 h despite diuretic use, persistently elevated (>2 mmol/L) lactate, metabolic acidosis with an increase (>4 mmol/L) in the base deficit, VIS at any point >20, and cardiac arrest or utilization of ECMO (extracorporeal membrane oxygenation) for hemodynamic instability within 48 h. Secondary outcomes included days on postoperative ventilation, development of acute kidney injury (AKI, defined as doubling of preoperative creatinine), cardiac arrest, ECMO use, CVICU length of stay (LOS), total postoperative LOS, and mortality.

### 2.2. Clinical and Surgical Management

Preoperative methylprednisolone (30 mg/kg) and heparin (400 U/kg, with a target ACT or 380 s) were administered to all Group 2 patients. Induction of anesthesia was performed in a standardized approach using ketamine, fentanyl, and rocuronium. Maintenance was achieved with fentanyl (2 µg/kg/h), dexmedetomidine (0.3 mg/kg/h), and sevoflurane titrated based on the anesthetic depth. The CPB circuit had a prime volume of 150–180 mL. Additional packed red blood cell (pRBC) transfusions were given to maintain a hematocrit of 30% or above during CPB and >40% prior to separation from CPB. CPB was performed with varying degrees of hypothermia based on individual metrics. Antegrade cold blood (20 mL/kg) was used to induce cardioplegia with an additional blood injection (10 mL/kg) if the aortic cross clamp (ACC) duration exceeded 50 min. Cardiopulmonary bypass pump flow was maintained at 100–150 mL/kg/min with mean arterial pressure targeted at 30–50 mmHg. Arteriovenous modified ultrafiltration was performed on all CPB patients for 15–20 min following separation from CPB. Approximately 10–20 mL/kg/min of fluid was removed from the arterial cannula, passed through the filter, and returned to the right atrium via the venous cannula. Following surgery, intravenous protamine sulphate was administered to neutralize heparin. All cases were admitted to CVICU for standard postoperative management by a dedicated team of pediatric intensivists with cardiac ICU training.

### 2.3. Sample Collection and Laboratory Protocol

**Figure 1:**
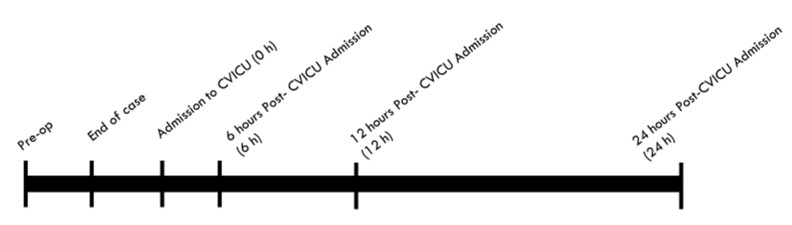
Sample collection timeline. CVICU = cardiovascular intensive care unit.

Blood was collected serially from a central arterial line during the perioperative period (Figure 1), immediately stored at 4 °C for less than 24 h until processing, and placed at −80 °C until analysis; these methods are demonstrated to minimize cytokine and histone loss in the clinical setting [24]. Circulating nucleosomes were measured from serum using a quantitative sandwich enzyme-linked immunosorbent assay (ELISA; Cell Death Detection ELISA^plus^ Kit, Version 14, Roche Diagnostics, Indianapolis, IN, USA) per the manufacturer’s instructions. Samples were run in duplicate and compared to the background and positive control. Circulating nucleosome levels are expressed as arbitrary units (AU).

Serum cytokines (interleukin-10 (IL-10)), IL-1α, IL-1β, IL-4, IL-6, IL-8, E-selectin, interferon-alpha (IFN-α), IFN-γ, IL-12 p70, IL-13, IL-17α, monocyte chemoattractant protein-1 (MCP-1), macrophage inflammatory protein-1 alpha (MIP-1α), MIP-1β, P-selectin, soluble cell adhesion molecule-1 (sICAM-1), C-X-C motif chemokine 10 (CXCL10), and tumor necrosis factor-alpha (TNF-α)) were measured using immunofluorescence technology (Custom ProcartaPlex™ Multiplex Immunoassay; Thermo Fisher, Waltham, MA, USA) per the manufacturer’s instructions. The plate was read on a Bio-Plex^®^ 200 array reader (Bio-Rad, Hercules, CA, USA). Samples were run in duplicate, and cytokine levels were quantified via comparison to a 4PL algorithm standard curve specific to each analyte.

### 2.4. Statistical Analysis

Categorical variables were tabulated as frequencies (%), and comparisons were achieved via chi-square or Fisher’s exact tests, as appropriate. Continuous variables were reported as means (± standard deviation (SD)), and comparisons utilized two-tailed Student’s t or Mann–Whitney *U* tests, as required. Correlations between continuous variables were evaluated with the Spearman test. Cytokine and nucleosome data were logarithmically transformed before analysis using log(x) (or log(x + 1) when values were <1) to reduce skew in data. Outliers were identified through the ROUT (robust regression and outlier removal) method, at a maximum false discovery rate of 0.1%, and excluded from the analysis. A linear mixed model repeated-measures analysis was used to examine the relationship between cytokine and histone levels and CPB or LCOS status. To establish the potential to predict LCOS development, receiver operating characteristic (ROC) curves were constructed for candidate clinical and laboratory variables previously identified as significantly different in bypass patients stratified by LCOS status. Cut-off points were determined using Youden’s J, and predictive ability was compared via area under the ROC curve (AUC). All results were considered significant when *p* < 0.05. Statistical analyses were performed with GraphPad Prism Version 6.07 (GraphPad Software, San Diego, CA, USA), with ROC curve metrics calculated by MedCalc Version 19.0.4 (MedCalc Software Ltd., Acacialaan, Belgium).

## 3. Results

### 3.1. Patient Demographics by Group

Of a total of thirty-two patients enrolled in the study (Figure S1), one patient was excluded from further analysis due to the use of ECMO intraoperatively for pulmonary hypertension, precluding the patient from further assessment for LCOS. Thirty-one patients (61.3% male), with a mean gestational age of 38.9 ± 0.58 weeks, completed the study (Table S1). The average age at the time of first (preoperative) sampling was 18.81 ± 25.37 days. No statistical differences in gender, gestational age at birth, age at first sample, or birth weight were found between groups.

### 3.2. Perioperative Variables

As expected, bypass patients were characterized by significantly higher (*p* = 0.0027) RACHS-1 classifications and EACTS-STS categories (*p* = 0.0022) as their heart defects were generally more complex than those of non-bypass patients (Table 1). PRISM III scores for 12 and 24 h did not differ between the groups, but the Group 2 average at 24 h was above 5 [25]. Bypass patients also had significantly higher PIM2 and SOFA scores at the time of CVICU admission, further underscoring the more serious nature of their heart defects.

There were no other significant differences in the measured through near-infrared spectroscopy (NIRS) at renal and bilateral cerebral hemispheres, hematocrit levels pre- or postoperatively, or preoperative ventilation.

### 3.3. Nucleosome and Cytokine Levels by Group

**Figure 2:**
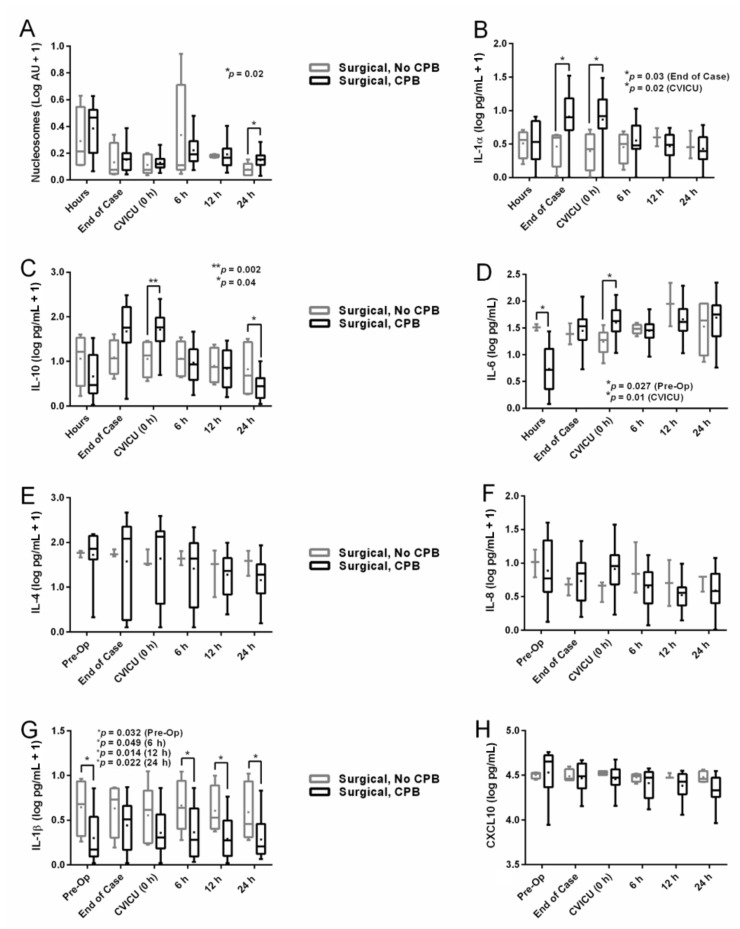
Serum cytokine and nucleosome levels (log-transformed) by group during perioperative period. (**A**), Circulating nucleosomes (log AU +1); *p* = 0.02 (24 h). (**B**), IL-1α (log pg/mL + 1); *p* = 0.03 (end of case) and *p* = 0.02 (CVICU). (**C**), IL-10 (log pg/mL + 1); *p* = 0.002 (CVICU) and *p* = 0.04 (24 h). (**D**), IL-6 (log pg/mL); *p* = 0.027 (pre-op) and *p* = 0.01 (CVICU). (**E**), IL-4 (log pg/mL + 1). (**F**), IL-8 (log pg/mL + 1). (**G**), IL-1β (log pg/mL + 1); *p* = 0.032 (pre-op), *p* = 0.049 (6 h), *p* = 0.014 (12 h), and *p* = 0.022 (24 h). (**H**), CXCL10 (log pg/mL). Whiskers extend to minimum and maximum values, box represents 25th to 75th percentiles, line indicates median, and + indicates mean. IL = interleukin, AU = arbitrary units, CPB = cardiopulmonary bypass, CVICU = cardiovascular intensive care unit, CXCL10 = C-X-C motif chemokine ligand 10, asterisks denote statistical significance.

To test our hypothesis that circulating nucleosomes are released in infants undergoing CPB, levels were serially measured and compared between the two groups. Notably, circulating nucleosome levels (Figure 2A) decreased in both groups from elevated preoperative levels, peaking postoperatively at 6 and 12 h in Group 2 and Group 1 patients, respectively. By 24 h postoperatively, Group 2 patients maintained elevated levels of nucleosomes, while levels in Group 1 patients were significantly lower. Levels of the cytokine IL-1α (Figure 2B) remained relatively stable in both groups except for end-of-case and CVICU admission timepoints, when levels spiked in bypass patients. In addition, levels of IL-10 (Figure 2C) followed a similar trend, with levels remaining relatively stable in Group 1 patients, but spiking in Group 2 patients at CVICU admission and plummeting 24 h after surgery. Levels of IL-6 (Figure 2D) varied widely in both groups during the perioperative period with no identifiable trend. However, Group 1 patients entered surgery with significantly higher IL-6 levels compared to bypass patients, but by CVICU admission, levels of IL-6 in bypass patients had spiked. Group 1 patients maintained higher levels of IL-1β (Figure 2G) across the entire perioperative period, resulting in four statistically elevated timepoints. Levels of MCP-1 (Figure S2G) and sICAM-1 (Figure S2K) were consistently higher in Group 1 patients, and significantly so at CVICU admission in sICAM-1 and 12 h in MCP-1. Levels of all remaining cytokines (Figure 2 and Figure S2) showed no significant differences between groups during the perioperative period. In all cases, a significant two-way nucleosome/cytokine × time interaction was noted on the status of CPB. Non-log-transformed values for Figure 2 nucleosomes and cytokines are provided in Figure S3.

Compared to Group 1 patients, bypass patients experienced longer postoperative ventilation, as well as longer CVICU and total postoperative length of stays. No patients required ECMO postoperatively, experienced cardiac arrest, or died (Table S2).

### 3.4. Patient Demographics Stratified by LCOS Status

The development of LCOS occurred in 11 of 26 bypass patients (Group 2, 42.31%), with a mean time of onset of 11.27 ± 4.43 h. No statistical differences in gender, gestational age at birth, age at first sample, or birth weight differentiated patients developing LCOS from those not developing the condition (Table 2). As anticipated, in the absence of CPB, no Group 1 patients developed LCOS.

### 3.5. Perioperative Variables Stratified by LCOS Status

Perioperative characteristics of bypass patients were classified by the presence or absence of LCOS and are noted in Table 3. The development of LCOS was not associated with differences in RACHS-1 classifications, EACTS-STS categories, PRISM III, PIM-2, or SOFA scores. Patients developing LCOS required significantly higher levels of inotropic medications at CVICU admission, and vasoactive-inotropic medications at 12 h postoperatively. Regional oxygenation, hematocrit, and pRBC also did not differ based on LCOS status. Preoperative ventilation was required in far fewer patients developing LCOS (9.09% vs. 46.67%, *p* = 0.04), potentially influencing secondary outcomes. Finally, patients developing LCOS required significantly longer aortic cross-clamp durations. While ACC duration and time on CPB were significantly correlated (ρ: 0.71, *p* < 0.0001), only ACC duration was associated with the development of LCOS.

### 3.6. Nucleosome and Cytokine Levels Stratified by LCOS Status

**Figure 3:**
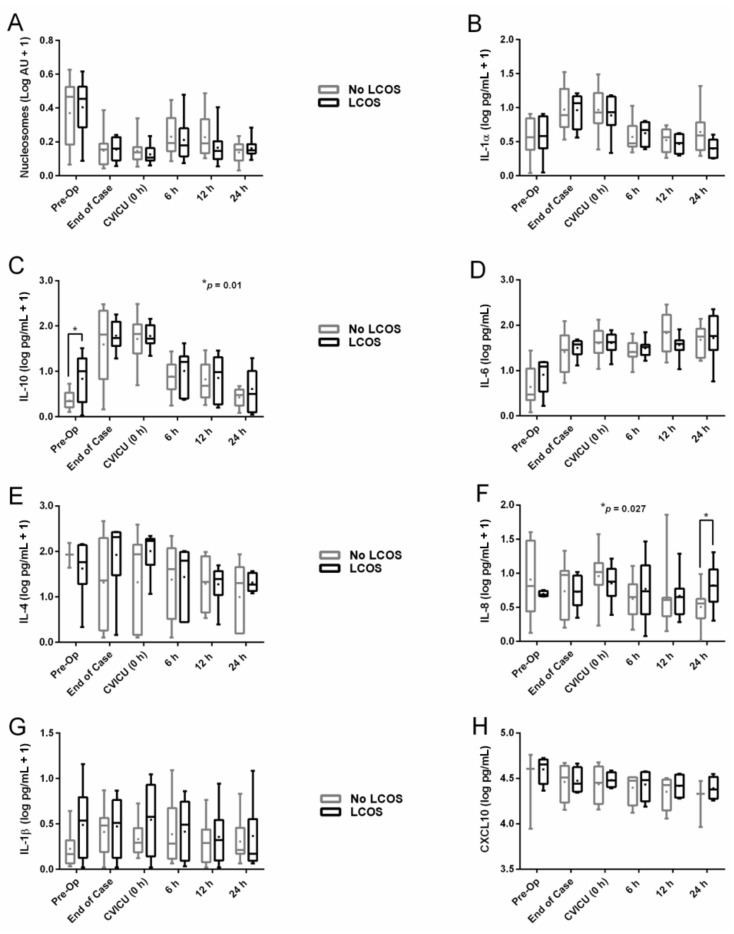
Group 2 serum cytokine and nucleosome levels (log-transformed) during the perioperative period stratified by LCOS status. (**A**), Circulating nucleosomes (log AU + 1). (**B**), IL-1α (log pg/mL + 1). (**C**), IL-10 (log pg/mL + 1); * *p* = 0.01 (pre-op). (**D**), IL-6 (log pg/mL). (**E**), IL-4 (log pg/mL + 1). (**F**), IL-8 (log pg/mL + 1); * *p* = 0.027 (24 h). (**G**), IL-1β (log pg/mL + 1). (**H**), CXCL10 (log pg/mL). Whiskers extend to minimum and maximum values, box represents 25th to 75th percentiles, line indicates median, and + indicates mean. LCOS = low cardiac output syndrome, IL = interleukin, AU = arbitrary units, CVICU = cardiovascular intensive care unit, CXCL10 = C-X-C motif chemokine ligand 10.

In all bypass patients, levels of IL-10 (Figure 3C) spiked from a relatively low level preoperatively to a peak during surgery, followed by a decline to near-baseline levels, irrespective of LCOS status. However, patients developing LCOS were characterized by significantly higher IL-10 levels preoperatively. Levels of IL-8 (Figure 3F) widely varied in all bypass patients during the perioperative period with no identifiable trend. However, by 24 h postoperatively, patients developing LCOS were characterized by significantly higher levels of IL-8. Patients developing LCOS also entered surgery with significantly higher levels of MIP-1α (Figure S4H), a chemokine released by macrophages. Levels of all remaining cytokines, as well as circulating nucleosomes, did not differ by LCOS status during the perioperative period (Figure 3 and Figure S4). In all cases, a significant two-way nucleosome/histone level x time interaction was noted on the primary outcome of LCOS. Non-log transformed values for Figure 3 nucleosomes and cytokines are provided in Figure S5.

### 3.7. Secondary Outcomes Stratified by LCOS Status

No secondary outcomes differed by LCOS status (Table 4). Counterintuitively, and potentially related to the higher incidence of preoperative ventilation in patients not developing LCOS, durations of postoperative ventilation, CVICU LOS, and total postoperative LOS were greater in patients not developing LCOS, though these trends did not reach statistical significance. Acute kidney injury occurred at a slightly higher incidence in patients developing LCOS (27.7% vs. 13.3%, *p* = 0.62), but this difference was not significant.

### 3.8. ROC Curve Analysis

**Figure 4:**
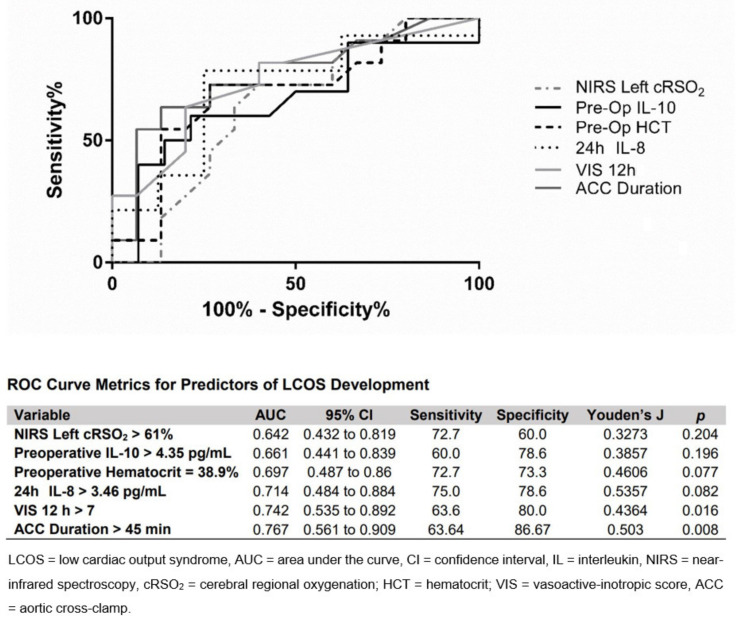
Performance of receiver operating characteristic (ROC) curves, representing the percentage of true positives (sensitivity) against the percentage of false positives (100% specificity), for clinical and laboratory variables potentially predicting the development of LCOS.

To evaluate the potential ability of surgical metrics or laboratory data to predict the development of LCOS in bypass patients, ROC curves were developed for several candidate variables showing discriminative ability in Figure 3 and Table 3, including NIRS Left cRSO_2_, preoperative IL-10 levels, preoperative hematocrit percentage, 24 h IL-8 levels, 12 h VIS, and duration of ACC. ROC curve analysis for ACC duration was moderately predictive of LCOS development with a cut-off of 45 min (AUC: 0.767; 95% confidence interval CI: 0.561 to 0.909; sensitivity: 63.64%; specificity 86.67%; *p* = 0.008; Figure 4). Though characterized by slightly lower AUCs, 24 h IL-8 levels (*p* = 0.082) and 12 h VIS (*p* = 0.016) ROC curves were also moderately predictive of LCOS development and not statistically different in predictive ability compared with ACC duration.

## 4. Discussion

We conducted this pilot study to determine whether any clinical metrics or perioperative changes in cytokine or circulating nucleosome levels in patients undergoing cardiac surgery could be of prognostic value for the development of LCOS. To our knowledge, this is the first study to suggest the surgical metric, aortic cross-clamp duration, may outperform more complex laboratory biomarker measurements in predicting the development of LCOS in infants undergoing CHD repair with CPB. Cross-clamping of the aorta induces myocardial ischemia, the effects of which are only somewhat mitigated through modern methods of cardioplegia, hypothermia, and hemodilution [26]. The biomechanical and metabolic stresses of this ischemia appear to be particularly pronounced in pediatric patients, where myocardial immaturity may create heightened responses [27]. In addition, longer aortic cross-clamping is often indicative of more complex surgical procedures, unanticipated perioperative complications, or difficulties weaning the patient from CPB [28], all of which increase the risk of poor postoperative outcomes, including the development of LCOS. While time on CPB is generally tightly correlated with ACC duration [29], in this study, there was no significant difference in CPB time based on LCOS status.

Recent studies have suggested circulating nucleosomes or extracellular histones may result in tissue damage through endothelial injury, platelet aggregation, coagulation activation, multisystem organ failure, and death [30,31,32]. Further, histones have been implicated in myocardial dysfunction associated with sepsis [33]. A previous study examining circulating nucleosome levels in infants with CHD demonstrated a correlation between peak postoperative nucleosome levels and poor outcomes, including prolonged mechanical ventilation and ICU LOS [18]. This information spurred the hypothesis that circulating nucleosomes may lead to a low cardiac output state and worsen patient outcomes in patients undergoing CPB. In comparison with Group 1 patients, Group 2 patients experienced elevated circulating nucleosomes during the entire perioperative period, but this was significant only at the 24 h timepoint. In addition, circulating nucleosome levels in Group 2 patients dropped precipitously from a preoperative peak, likely due to the administration of histone-binding heparin during CPB [34]. This is in line with previous studies revealing that heparin, as a highly negatively charged molecule, binds to histones and prevents its cytotoxicity [35,36]. Circulating nucleosome levels in bypass patients, however, did not differ based on LCOS status.

The systemic inflammatory response induced by cardiopulmonary bypass has been documented in pediatric patients through elevations of both pro- and anti-inflammatory cytokines [7,9,11,12,13]. Elevations in cytokine levels are often still evident in bypass patients despite the standard and intentional use of perioperative steroids to blunt this inflammatory response [37]. Here, despite universal administration of preoperative steroids in Group 2 patients, bypass induced significantly higher levels of IL-1α, IL-10, and IL-6 compared with Group 1 patients at admission to the CVICU, but these differences waned by the 6 h mark. Interestingly, levels of IL-1α only differentiate Group 2 patients from Group 1 during and immediately following surgery, with elevations of IL-1α potentially representing a compensatory response to myocardial ischemia [38]. Within Group 2 patients, the development of LCOS was only associated postoperatively with elevated IL-8 levels at the 24 h mark. Preoperatively, patients later developing LCOS were characterized by higher IL-10 and MIP-1α levels. Interestingly, early elevation of IL-10 levels has been associated with poor prognosis in several serious conditions [39,40,41,42], potentially warranting further study in the infant CPB population. However, cytokine differences differentiated by LCOS status were not able to predict the development of LCOS with much accuracy, potentially due to variability in administration of inotropic medications, agents that are known to influence cytokine levels [43]. Additional influences of heparin or protamine sulfate on cytokine levels cannot be ruled out.

Patients most frequently met the criteria for LCOS determination due to hypotension and/or oliguria and metabolic acidosis at an average postoperative duration of 11.27 h. This timepoint is within the typically reported window of LCOS development [1]. Ninety-two percent of Group 2 patients are considered developmentally neonatal. The 42% reported incidence of LCOS in this study correlates well with prior literature in predominately or exclusively neonatal cardiac patients [44,45], indicating our parameters for the diagnosis of the often controversially defined LCOS were likely appropriate. Additionally, the high RACHS-1 categorizations of our Group 2 patients (76.9% ≥ Category 4), consistent with complex operations, likely contributed to our relatively high LCOS incidence. Given that vasoactive and inotropic medications are often supplied in response to, or anticipation of, LCOS, VIS was, unsurprisingly, higher in patients developing LCOS, but statistically significant only at 12 h postoperatively. A high VIS (>15) has been associated with increased morbidity and mortality in a similar population of infants undergoing cardiac surgery [23], but patients developing LCOS in this study barely scored >10 at 24 h. While studies have demonstrated inconsistent associations between the development of LCOS and prolonged mechanical ventilation [3,44], CVICU LOS, and total postoperative LOS, our LCOS patients trended toward fewer postoperative ventilation days and shorter CVICU and total postoperative LOS. This lack of significant differences in secondary outcomes is likely attributable to the significantly higher number of non-LCOS patients requiring preoperative mechanical ventilation (46.67 vs. 9.09%, *p* = 0.04), potentially indicating additional health complexities predating cardiac surgery. Mechanical ventilation has been shown to induce inflammatory lung injury resulting in elevated histone levels [46], so the high rate of preoperative ventilation in non-LCOS patients may also have influenced cytokine and circulating nucleosome levels, potentially masking differences in these populations.

The results of this study are subject to several limitations. The study design was based on a single site, resulting in a particularly complex and heterogeneous patient population frequently requiring CPB. The findings here represent those of patients at one center with one surgical approach and therefore cannot be extrapolated to other centers with different patient populations and surgical approaches. Furthermore, the small sample size among all the groups, specifically the patients who underwent cardiac surgery with no bypass, is a limitation in our study and could have affected our results.

An additional limitation is the use of the clinical diagnostic criteria for LCOS without invasive cardiac output monitoring, which could potentially influence our results. Another limitation is the use of cytokine measurements as a proxy for processes involved in systemic inflammation. Cytokines and chemokines have short half-lives and, in the case of chemokines in particular, act on local cell populations, often resulting in low concentrations in peripheral blood. The short-lived nature of these biomarkers means they provide only a narrow glimpse of the inflammatory status of a patient, but measurements at six time points in this study should serve to counteract this temporal variability. Additionally, while attempts were made to limit variability in patient, clinical, and surgical management, potential confounders influencing cytokine and circulating nucleosome concentrations, such as ultrafiltrate volume, the volume of cardioplegic solution, use of inhaled nitric oxide, transfused blood volume, or dosages of inotropic medications, are impossible to eliminate.

## 5. Conclusions

The use of cardiopulmonary bypass was associated with postoperative elevations in circulating nucleosomes, IL-6, IL-10, and IL-1α levels, as well as longer postoperative ventilation, CVICU LOS, and total postoperative LOS compared to cardiac surgery in infants without CPB. Bypass patients developing LCOS were characterized by elevated preoperative IL-10 and MIP-1α levels, as well as elevated 24 h IL-8 levels, but they did not differ in recorded outcomes from bypass patients not developing LCOS. Further, differences identified through laboratory biomarker measurements were insufficient compared to the surgical metric of aortic cross-clamp duration in predicting the development of LCOS. Larger studies may be warranted to further characterize population differences associated with the development of LCOS.

## Figures and Tables

**Table 1:** Perioperative variables by group.

Perioperative Characteristic	Group 1 (*n* = 5)	Group 2 (*n* = 26)	*p*
RACHS-1, Category 1–3, *n* (%) RACHS-1, Category 4–6, *n* (%)	5 (100) 0 (0)	6 (23.1) 20 (76.9)	0.0027
STS–EACTS, Category 1–2, *n* (%)	4 (80)	2 (7.7)	0.0022
STS–EACTS, Category 3–5, *n* (%)	1 (20)	24 (92.3)	
PRISM III Score, 12 h mean ± SD	3.83 ± 3.15	4.64 ± 3.97	0.7452
PRISM III Score, 24 h, mean ± SD	4.38 ± 2.89	6.79 ± 8	0.7434
PIM2 Score, CVICU, mean ± SD	1.45 ± 1.13	4.63 ± 3.07	0.003
SOFA Score, CVICU, mean ± SD	8.50 ± 1.29	10.63 ± 1.79	0.0342
SOFA Score, 12 h, mean ± SD	6.60 ± 3.98	9.52 ± 1.75	0.0691
SOFA Score, 24 h, mean ± SD	5.67 ± 5.13	9.06 ± 2.26	0.2639
VIS, CVICU, mean ± SD	0.70 ± 1.095	8.64 ± 3.51	<0.0001
IS, CVICU, mean ± SD	0.00 ± 0.00	1.37 ± 1.26	0.0298
VIS, 12 h, mean ± SD	1.70 ± 2.64	7.40 ± 2.80	0.0012
IS, 12 h, mean ± SD	0.00 ± 0.00	1.12 ± 1.24	0.0813
VIS, 24 h, mean ± SD	1.30 ± 1.86	8.74 ± 3.39	<0.0001
IS, 24 h, mean ± SD	0.00 ± 0.00	1.15 ± 1.43	0.1154
NIRS Left cRSO_2_, CVICU, %, mean ± SD	74.4 ± 11.52	62.62 ± 14.3	0.1056
NIRS Left cRSO_2_, 12 h, %, mean ± SD	73.2 ± 12.72	61.58 ± 12.66	0.0729
NIRS Left cRSO_2_, 24 h, %, mean ± SD	62.5 ± 14.85	64.54 ± 14.15	0.8175
NIRS Right cRSO_2_, CVICU, %, mean ± SD	75.5 ± 22.22	57.77 ± 17.91	0.1806
NIRS Right cRSO_2_, 12 h, %, mean ± SD	64.5 ± 18.52	60.42 ± 14.06	0.3658
NIRS Right cRSO_2_, 24 h, %, mean ± SD	57 ± 35.36	62.73 ± 14.72	>0.9999
NIRS rRSO_2_, CVICU, %, mean ± SD	74.2 ± 12.24	77.85 ± 16.02	0.4877
NIRS rRSO_2_, 12 h, %, mean ± SD	79.8 ± 19.68	76.58 ± 14.34	0.4553
NIRS rRSO_2_, 24 h, %, mean ± SD	75 ± 28.28	72.12 ± 12.78	0.963
Preoperative HCT, %, mean ± SD	44.88 ± 4.77	40.35 ± 5.4	0.0732
Postoperative HCT, %, mean ± SD	38 ± 7.38	36.56 ± 5.35	0.7251
pRBC, mL/kg, mean ± SD	8.2 ± 11.23	19.92 ± 19.46	0.2348
Preoperative Ventilation, *n* (%)	1 (20)	8 (30.77)	0.6271

RACHS-1 = Risk Adjustment for Congenital Heart Surgery, STS-EACTS mortality Categories = European Association for Cardiothoracic Surgery and the Society of Thoracic Surgeons, PRISM = Pediatric Risk of Mortality, SD = standard deviation, PIM2 = Pediatric Index of Mortality 2, CVICU = cardiovascular intensive care unit, SOFA = Sequential Organ Failure Assessment, VIS = vasoactive-inotropic score, IS = inotropic score, NIRS = near-infrared spectroscopy, cRSO_2_ = cerebral regional oxygenation, rRSO_2_ = renal regional oxygenation, HCT = hematocrit, pRBC = packed red blood cell volume.

**Table 2:** Group 2 demographics stratified by primary outcome.

Demographics	No LCOS (*n* = 15)	LCOS (*n* = 11)	*p*
Sex, male, *n* (%)	9 (60)	7 (63.6)	1.000
GA at Birth, weeks, mean ± SD	38.83 ± 0.54	38.9 ± 0.66	0.598
Age at First Sample, days, mean ± SD	20.20 ± 22.49	21.36 ± 34	0.298
Birth Weight, kg, mean ± SD	3.38 ± 0.63	3.45 ± 0.8	0.949
Type of CHD	HLHS	HLHS	
	COA	IAA and VSD	
	DORV	DORV, PA	
	COA, ASD, VSD, TA	D-TGA	
	TOF	COA and AS	
	TA, ASD, VSD	COA and ASD	
	DORV, TGA, VSD, COA		
	DORV, TOF, PA		

LCOS = low cardiac output syndrome, GA = gestational age, SD = standard deviation, COA = coarctation of the aorta, TA = tricuspid valve atresia, VSD = ventricular septal defect, HLHS = hypoplastic heart syndrome, IAA = interrupted aortic arch, ASD = atrial septal defect, DORV = double outlet right ventricle, PA = pulmonary atresia, D-TGA = dextro-transposition of the great arteries, AS = aortic valve stenosis, TOF = tetralogy of Fallot, CHD = congenital heart disease.

**Table 3:** Group 2 perioperative variables stratified by primary outcome.

Perioperative Characteristic	No LCOS (*n* = 15)	LCOS (*n* = 11)	*p*
RACHS-1, Category 1–3, *n* (%)	4 (26.67)	2 (18.18)	1.000
RACHS-1, Category 4–6, *n* (%)	11 (73.33)	9 (81.81)	
STS–EACTS, Category 1–2, *n* (%)	1 (6.7)	0(0)	1.000
STS–EACTS, Category 3–5, *n* (%)	14 (99.3)	100 (100)	
PRISM III Score, 12 h, mean ± SD	4.86 ± 4.59	4.32 ± 3.12	0.788
PRISM III Score, 24 h, mean ± SD	7.7 ± 10.19	5.56 ± 3.43	0.73
PIM2 Score, CVICU, mean ± SD	4.85 ± 3.04	4.32 ± 3.23	0.646
SOFA Score, CVICU, mean ± SD	10.23 ± 2.09	11.09 ± 1.3	0.347
SOFA Score, 12 h, mean ± SD	9.4 ± 1.58	9.64 ± 1.96	0.588
SOFA Score, 24 h, mean ± SD	9.44 ± 2.19	8.67 ± 2.4	0.473
VIS, CVICU, mean ± SD	8.57 ± 3.82	8.73 ± 3.23	0.745
IS, CVICU, mean ± SD	1.00 ± 1.25	1.87 ± 1.14	0.0826
VIS, 12 h, mean ± SD	6.4 ± 1.97	8.77 ± 3.26	0.032
IS, 12 h, mean ± SD	0.93 ± 1.28	1.36 ± 1.21	0.292
VIS, 24 h, mean ± SD	7.87 ± 3.49	10.05 ± 2.93	0.061
IS, 24 h, mean ± SD	1.13 ± 1.46	1.18 ± 1.47	0.947
NIRS Left cRSO_2_, CVICU, %, mean ± SD	59.8 ± 15.89	66.45 ± 11.4	0.232
NIRS Left cRSO_2_, 12 h, %, mean ± SD	62.53 ± 14.2	60.27 ± 10.72	0.637
NIRS Left cRSO_2_, 24 h, %, mean ± SD	63.47 ± 16.54	66 ± 10.65	0.583
NIRS Right cRSO_2_, CVICU, %, mean ± SD	54 ± 20.87	62.91 ± 11.89	0.391
NIRS Right cRSO_2_, 12 h, %, mean ± SD	59.87 ± 16.81	61.18 ± 9.87	0.919
NIRS Right cRSO_2_, 24 h, %, mean ± SD	61.13 ± 18.28	64.91 ± 8.06	0.909
NIRS rRSO_2_, CVICU, %, mean ± SD	74.8 ± 19.25	82 ± 9.5	0.481
NIRS rRSO_2_, 12 h, %, mean ± SD	74.67 ± 12.78	79.18 ± 16.51	0.264
NIRS rRSO_2_, 24 h, %, mean ± SD	71.07 ± 13.74	73.55 ± 11.82	0.655
Preoperative HCT, %, mean ± SD	41.79 ± 5.76	38.39 ± 4.38	0.094
Postoperative HCT, %, mean ± SD	37.9 ± 5.48	34.98 ± 4.99	0.191
pRBC, mL/kg, mean ± SD	15.36 ± 14.56	26.15 ± 23.99	0.296
Preoperative Ventilation, *n* (%)	7 (46.67)	1 (9.09)	0.04
CPB Duration, min, mean ± SD	100.9 ± 25.9	111.9 ± 26.13	0.253
ACC Duration, min, mean ± SD	26.87 ± 18.22	45.55 ± 21.53	0.021

LCOS = low cardiac output syndrome, RACHS-1 = Risk Adjustment for Congenital Heart Surgery, STS-EACTS = the Society of Thoracic Surgeons and European Association for Cardiothoracic Surgery, PRISM = Pediatric Risk of Mortality, SD = standard deviation, PIM2 = Pediatric Index of Mortality 2, CVICU = cardiovascular intensive care unit, SOFA = Sequential Organ Failure Assessment, VIS = vasoactive-inotropic score, IS = inotropic score, NIRS = near-infrared spectroscopy, cRSO_2_ = cerebral regional oxygenation, rRSO_2_ = renal regional oxygenation, HCT = hematocrit, pRBC = packed red blood cell volume, CPB = cardiopulmonary bypass, ACC = aortic cross-clamp.

**Table 4:** Group 2 secondary outcomes stratified by primary outcome.

Secondary Outcome	No LCOS (*n* = 15)	LCOS (*n* = 11)	*p*
Postoperative Ventilation, days, mean ± SD	8.53 ± 14.98	3.91 ± 1.7	0.305
AKI, *n* (%)	2 (13.3)	3 (27.7)	0.62
CVICU LOS, days, mean ± SD	14.93 ± 18.83	7.36 ± 2.01	0.18
Total Postoperative LOS, days, mean ± SD	44 ± 38.37	27.09 ± 16.7	0.35

LCOS = low cardiac output syndrome, AKI = acute kidney injury, CVICU = cardiovascular intensive care, LOS = length of stay.

## Data Availability

All data are available in the study and are available upon request.

## Supplementary Materials

The following are available online at https://www.mdpi.com/2077-0383/10/4/712/s1, Figure S1. Patient enrollment; Figure S2. Additional cytokine levels by group; Figure S3. Cytokine levels by group non-log transformed; Figure S4. Additional cytokine levels by outcome; Figure S5. Cytokine levels by outcome non-log transformed; Table S1. Patient demographics by group; Table S2. Secondary outcomes by group.

## References

[1] Chandler H.K., Kirsch R. (2016). Management of the Low Cardiac Output Syndrome Following Surgery for Congenital Heart Disease. Curr. Cardiol. Rev..

[2] Seghaye M., Engelhardt W., Grabitz R., Faymonville M., Hörnchen H., Messmer B., Von Bernuth G. (1993). Multiple System Organ Failure after Open Heart Surgery in Infants and Children. Thorac. Cardiovasc. Surg..

[3] Hoffman T.M., Wernovsky G., Atz A.M., Kulik T.J., Nelson D.P., Chang A.C., Bailey J.M., Akbary A., Kocsis J.F., Kaczmarek R. (2003). Efficacy and Safety of Milrinone in Preventing Low Cardiac Output Syndrome in Infants and Children after Corrective Surgery for Congenital Heart Disease. Circulation.

[4] Schroeder V.A., Pearl J.M., Schwartz S.M., Shanley T.P., Manning P.B., Nelson D.P. (2003). Combined Steroid Treatment for Congenital Heart Surgery Improves Oxygen Delivery and Reduces Postbypass Inflammatory Mediator Expression. Circulation.

[5] Zakkar M., Guida G., Suleiman M.-S., Angelini G.D. (2015). Cardiopulmonary Bypass and Oxidative Stress. Oxidative Med. Cell. Longev..

[6] Seghaye M.-C. (2003). The clinical implications of the systemic inflammatory reaction related to cardiac operations in children. Cardiol. Young.

[7] Carmona F., Manso P.H., Vicente W.V., Castro M., Carlotti A.P. (2008). Risk stratification in neonates and infants submitted to cardiac surgery with cardiopulmonary bypass: A multimarker approach combining inflammatory mediators, N-terminal pro-B-type natriuretic peptide and troponin I. Cytokine.

[8] Robert S.M., Borasino S., Dabal R.J., Cleveland D.C., Hock K.M., Alten J.A. (2015). Postoperative Hydrocortisone Infusion Reduces the Prevalence of Low Cardiac Output Syndrome after Neonatal Cardiopulmonary Bypass*. Pediatr. Crit. Care Med..

[9] Mahle W.T., Matthews E., Kanter K.R., Kogon B.E., Hamrick S.E., Strickland M.J. (2014). Inflammatory Response after Neonatal Cardiac Surgery and Its Relationship to Clinical Outcomes. Ann. Thorac. Surg..

[10] Kozik D.J., Tweddell J.S. (2006). Characterizing the Inflammatory Response to Cardiopulmonary Bypass in Children. Ann. Thorac. Surg..

[11] Allan C.K., Newburger J.W., McGrath E., Elder J., Psoinos C., Laussen P.C., Del Nido P.J., Wypij D., McGowan F.X. (2010). The Relationship between Inflammatory Activation and Clinical Outcome after Infant Cardiopulmonary Bypass. Anesthesia Analg..

[12] Appachi E., Mossad E., Mee R.B., Bokesch P. (2007). Perioperative Serum Interleukins in Neonates with Hypoplastic Left-Heart Syndrome and Transposition of the Great Arteries. J. Cardiothorac. Vasc. Anesthesia.

[13] Hövels-Gürich H.H., Vazquez-Jimenez J.F., Silvestri A., Schumacher K., Minkenberg R., Duchateau J., Messmer B.J., Von Bernuth G., Seghaye M.-C. (2002). Production of proinflammatory cytokines and myocardial dysfunction after arterial switch operation in neonates with transposition of the great arteries. J. Thorac. Cardiovasc. Surg..

[14] Zeerleder S., Zwart B., Wuillemin W.A., Aarden L.A., Groeneveld A.B.J., Caliezi C., Van Nieuwenhuijze A.E.M., Van Mierlo G.J., Eerenberg A.J.M., Lämmle B. (2003). Elevated nucleosome levels in systemic inflammation and sepsis. Crit. Care Med..

[15] Beaubien-Souligny W., Neagoe P.-E., Gagnon D., Denault A.Y., Sirois M.G. (2020). Increased Circulating Levels of Neutrophil Extracellular Traps during Cardiopulmonary Bypass. CJC Open.

[16] Savchenko A.S., Borissoff J.I., Martinod K., De Meyer S.F., Gallant M., Erpenbeck L., Brill A., Wang Y., Wagner D.D. (2014). VWF-mediated leukocyte recruitment with chromatin decondensation by PAD4 increases myocardial ischemia/reperfusion injury in mice. Blood.

[17] Qi Y., Uchida T., Yamamoto M., Yamamoto Y., Kido K., Ito H., Ohno N., Asahara M., Yamada Y., Yamaguchi O. (2016). Perioperative Elevation in Cell-Free DNA Levels in Patients Undergoing Cardiac Surgery: Possible Contribution of Neutrophil Extracellular Traps to Perioperative Renal Dysfunction. Anesthesiol. Res. Pr..

[18] Gao H., Zhang N., Lu F., Yu X., Zhu L., Mo X., Wang W. (2016). Circulating histones for predicting prognosis after cardiac surgery: A prospective study. Interact. Cardiovasc. Thorac. Surg..

[19] O’Brien S.M., Clarke D.R., Jacobs J.P., Jacobs M.L., Lacour-Gayet F.G., Pizarro C., Welke K.F., Maruszewski B., Tobota Z., Miller W.J. (2009). An empirically based tool for analyzing mortality associated with congenital heart surgery. J. Thorac. Cardiovasc. Surg..

[20] Slater A., Shann F., Pearson G., for the PIM Study Group (2003). PIM2: A revised version of the Paediatric Index of Mortality. Intensiv. Care Med..

[21] Pollack M.M., Patel K.M., Ruttimann U.E. (1996). PRISM III. Crit. Care Med..

[22] Vincent J.-L., De Mendonca A., Cantraine F., Moreno R., Takala J., Suter P.M., Sprung C.L., Colardyn F., Blecher S. (1998). Use of the SOFA score to assess the incidence of organ dysfunction/failure in intensive care units. Crit. Care Med..

[23] Gaies M.G., Gurney J.G., Yen A.H., Napoli M.L., Gajarski R.J., Ohye R.G., Charpie J.R., Hirsch J.C. (2010). Vasoactive–inotropic score as a predictor of morbidity and mortality in infants after cardiopulmonary bypass*. Pediatr. Crit. Care Med..

[24] Aziz N., Detels R., Quint J.J., Li Q., Gjertson D., Butch A.W. (2016). Stability of cytokines, chemokines and soluble activation markers in unprocessed blood stored under different conditions. Cytokine.

[25] Tyagi P., Agrawal M., Tullu M.S. (2018). Comparison of Pediatric Risk of Mortality III, Pediatric Index of Mortality 2, and Pediatric Index of Mortality 3 in Predicting Mortality in a Pediatric Intensive Care Unit. J. Pediatr. Intensiv. Care.

[26] Baikoussis N.G., Papakonstantinou N.A., Verra C., Kakouris G., Chounti M., Hountis P., Dedeilias P., Argiriou M. (2015). Mechanisms of oxidative stress and myocardial protection during open-heart surgery. Ann. Card. Anaesth..

[27] Hasegawa T., Yamaguchi M., Yoshimura N., Okita Y. (2005). The dependence of myocardial damage on age and ischemic time in pediatric cardiac surgery. J. Thorac. Cardiovasc. Surg..

[28] Onorati F., De Feo M., Mastroroberto P., Cristodoro L., Pezzo F., Renzulli A., Cotrufo M. (2005). Determinants and Prognosis of Myocardial Damage After Coronary Artery Bypass Grafting. Ann. Thorac. Surg..

[29] Nissinen J., Biancari F., Wistbacka J.-O., Peltola T., Loponen P., Tarkiainen P., Virkkilä M., Tarkka M. (2009). Safe time limits of aortic cross-clamping and cardiopulmonary bypass in adult cardiac surgery. Perfusion.

[30] Fuchs T.A., Bhandari A.A., Wagner D.D. (2011). Histones induce rapid and profound thrombocytopenia in mice. Blood.

[31] Xu J., Zhang X., Monestier M., Esmon N.L., Esmon C.T. (2011). Extracellular Histones Are Mediators of Death through TLR2 and TLR4 in Mouse Fatal Liver Injury. J. Immunol..

[32] Allam R., Scherbaum C.R., Darisipudi M.N., Mulay S.R., Hägele H., Lichtnekert J., Hagemann J.H., Rupanagudi K.V., Ryu M., Schwarzenberger C. (2012). Histones from Dying Renal Cells Aggravate Kidney Injury via TLR2 and TLR4. J. Am. Soc. Nephrol..

[33] Kalbitz M., Grailer J.J., Fattahi F., Jajou L., Herron T.J., Campbell K.F., Zetoune F.S., Bosmann M., Sarma J.V., Huber-Lang M. (2015). Role of extracellular histones in the cardiomyopathy of sepsis. FASEB J..

[34] Hogwood J., Gray E., Komorowicz E., Varjú I., Varga Z., Kolev K.N., Longstaff C. (2016). Neutralisation of the anti-coagulant effects of heparin by histones in blood plasma and purified systems. Thromb. Haemost..

[35] Zhu C., Liang Y., Li X., Chen N., Ma X. (2019). Unfractionated heparin attenuates histone-mediated cytotoxicity in vitro and prevents intestinal microcirculatory dysfunction in histone-infused rats. J. Trauma Acute Care Surg..

[36] Wildhagen K.C.A.A., De Frutos P.G., Reutelingsperger C.P., Schrijver R., Aresté C., Ortega-Gómez A., Deckers N.M., Hemker H.C., Soehnlein O., Nicolaes G.A.F. (2014). Nonanticoagulant heparin prevents histone-mediated cytotoxicity in vitro and improves survival in sepsis. Blood.

[37] Hauser G.J., Ben-Ari J., Colvin M.P., Dalton H.J., Hertzog J.H., Bearb M., Hopkins R.A., Walker S.M. (1998). Interleukin-6 levels in serum and lung lavage fluid of children undergoing open heart surgery correlate with postoperative morbidity. Intensiv. Care Med..

[38] Salmeron K.E., Maniskas M.E., Edwards D.N., Wong R., Rajkovic I., Trout A., Rahman A.A., Hamilton S., Fraser J.F., Pinteaux E. (2019). Interleukin 1 alpha administration is neuroprotective and neuro-restorative following experimental ischemic stroke. J. Neuroinflamm..

[39] Cunha L.L., Morari E.C., Nonogaki S., Marcello M.A., Soares F.A., Vassallo J., Ward L.S. (2016). Interleukin 10 expression is related to aggressiveness and poor prognosis of patients with thyroid cancer. Cancer Immunol. Immunother..

[40] Hempel L., Körholz D., Nusbaum P., Bönig H., Burdach S., Zintl F. (1997). High interleukin-10 serum levels are associated with fatal outcome in patients after bone marrow transplantation. Bone Marrow Transplant..

[41] Welsh P., Murray H.M., Ford I., Trompet S., De Craen A.J., Jukema J.W., Stott D.J., McInnes I.B., Packard C.J., Westendorp R.G. (2011). Circulating Interleukin-10 and Risk of Cardiovascular Events. Arter. Thromb. Vasc. Biol..

[42] Szaflarska A., Szczepanik A., Siedlar M., Czupryna A., Sierzega M., Popiela T., Zembala M. (2009). Preoperative plasma level of IL-10 but not of proinflammatory cytokines is an independent prognostic factor in patients with gastric cancer. Anticancer Res..

[43] Matsumori A. (1997). The use of cytokine inhibitors. Int. J. Cardiol..

[44] Butts R.J., Scheurer M.A., Atz A.M., Zyblewski S.C., Hulsey T.C., Bradley S.M., Graham E.M. (2012). Comparison of Maximum Vasoactive Inotropic Score and Low Cardiac Output Syndrome as Markers of Early Postoperative Outcomes after Neonatal Cardiac Surgery. Pediatr. Cardiol..

[45] Graham E.M., Atz A.M., Butts R.J., Baker N.L., Zyblewski S.C., Deardorff R.L., DeSantis S.M., Reeves S.T., Bradley S.M., Spinale F.G. (2011). Standardized preoperative corticosteroid treatment in neonates undergoing cardiac surgery: Results from a randomized trial. J. Thorac. Cardiovasc. Surg..

[46] Yildiz C., Palaniyar N., Otulakowski G., Khan M.A., Post M., Kuebler W.M., Tanswell K., Belcastro R., Masood A., Engelberts D. (2015). Mechanical Ventilation Induces Neutrophil Extracellular Trap Formation. Anesthesiology.

