# Supplementary material for: Clinical and Laboratory Predictors for the Development of Low Cardiac Output Syndrome in Infants Undergoing Cardiopulmonary Bypass: A Pilot Study
Source: J Clin Med. 2021 Feb 11;10(4):712. doi: 10.3390/jcm10040712 (PMC7916966; doi:10.3390/jcm10040712)
Supplement: Supplementary file 1 [file jcm-10-00712-s001.zip › Suppl file/Supplemental Table 2.docx]

**Table 2. Secondary Outcomes by Group**

| **Secondary Outcome** | **Group 1 (*n* = 5)** | **Group 2 (*n* = 26)** | ***p*** |
| --- | --- | --- | --- |
| **Postoperative Ventilation, days, mean ± SD** | 1.4 ± 1.67 | 6.58 ± 11.5 | **0.0062** |
| **AKI, n (%)** | 0 (0) | 5 (19.23) | 0.5601 |
| **CVICU LOS, days, mean ± SD** | 7.4 ± 11.52 | 11.73 ± 14.65 | **0.0312** |
| **Total Postoperative LOS, days, mean ± SD** | 14.8 ± 16.07 | 36.85 ± 31.76 | **0.0317** |

SD = standard deviation, AKI = acute kidney injury, CVICU = cardiovascular intensive care, LOS = length of stay.
