# Supplementary material for: Clinical and Laboratory Predictors for the Development of Low Cardiac Output Syndrome in Infants Undergoing Cardiopulmonary Bypass: A Pilot Study
Source: J Clin Med. 2021 Feb 11;10(4):712. doi: 10.3390/jcm10040712 (PMC7916966; doi:10.3390/jcm10040712)
Supplement: Supplementary file 1 [file jcm-10-00712-s001.zip › Suppl file/Supplementary Table 1.docx]

**Supplementary Table 1. Patient Demographics by Group**

| **Demographics** | **Group 1 (*n* = 5)** | **Group 2 (*n* = 26)** | ***p*** | |
| --- | --- | --- | --- | --- |
| **Sex, male, n (%)** | 3 (60) | 16 (62) | | >0.999 |
| **GA at Birth, wk, mean ± SD** | 39.0 ± 0.65 | 38.86 ± 0.58 | | 0.866 |
| **Age at First Sample, days, mean ± SD** | 9 ± 4.58 | 20.69 ± 27.31 | | 0.25 |
| **Birth Weight, kg, mean ± SD** | 3.42 ± 0.33 | 3.41 ± 0.69 | | 0.334 |
| **Type of CHD** | COA  TA/NRGA, VSD | HLHS  IAA and VSD  DORV, PA  DORV  COA, ASD, VSD, TA  D-TGA  COA and AS  TOF  DORV, TGA, VSD  COA, ASD  DORV, TOF, PA | |  |

GA = gestational age, SD = standard deviation, COA = coarctation of the aorta, TA = tricuspid atresia, VSD = Ventricular septal defect, HLHS = hypoplastic heart syndrome, IAA = interrupted aortic arch, ASD = Atrial septal defect, DORV = double outlet right ventricle, PA = pulmonary atresia, TA = tricuspid valve atresia, D-TGA = dextro-transposition of the great arteries, AS= aortic valve stenosis, TOF = tetralogy of fallot.
