# Supplementary figures and images for: Clinical and Laboratory Predictors for the Development of Low Cardiac Output Syndrome in Infants Undergoing Cardiopulmonary Bypass: A Pilot Study
Source: J Clin Med. 2021 Feb 11;10(4):712. doi: 10.3390/jcm10040712 (PMC7916966; doi:10.3390/jcm10040712)

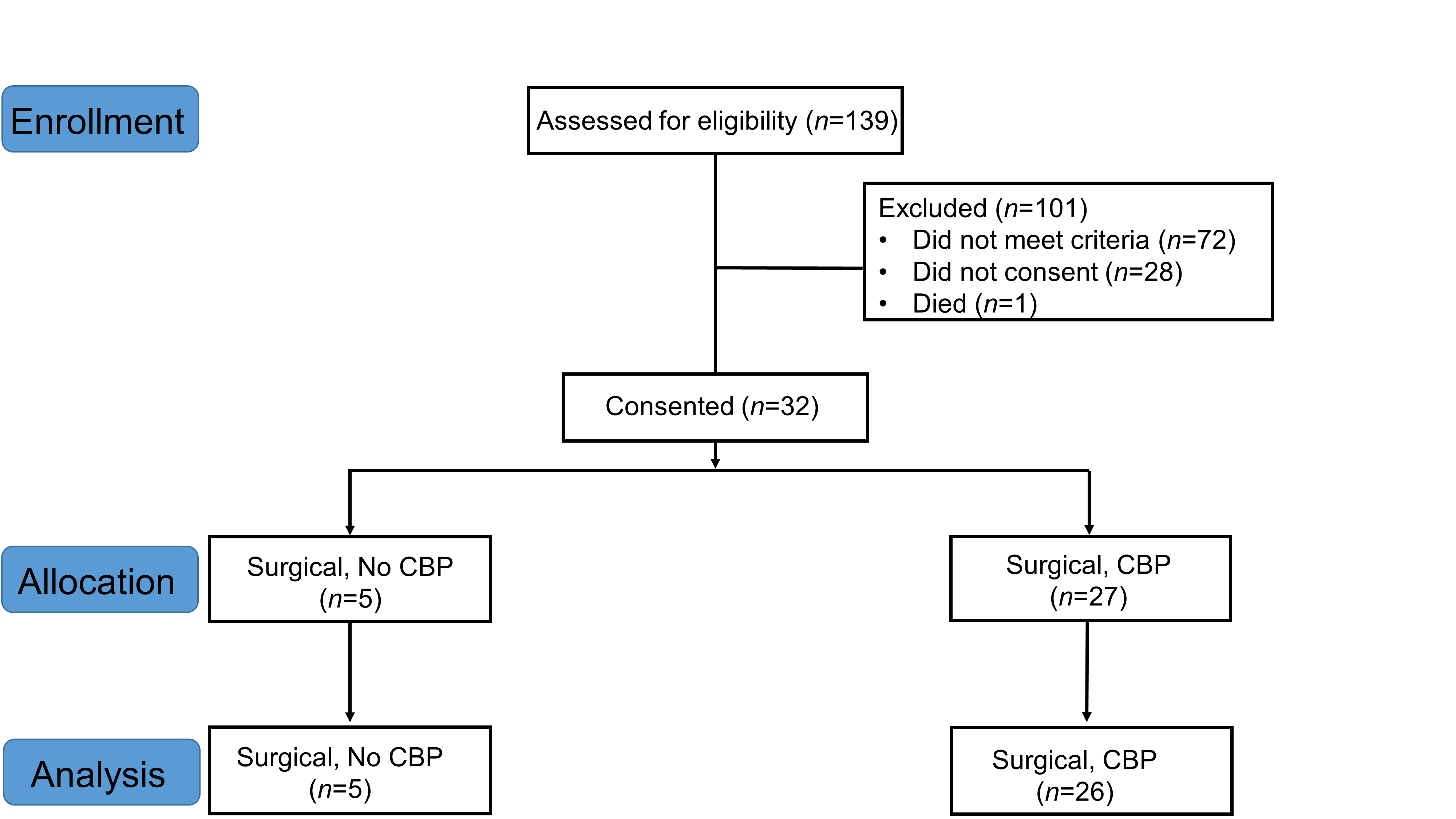

Supplement: Supplementary file 1 [file jcm-10-00712-s001.zip › Suppl file/Supplementary Figure 1.tif]
